# Inhibiting tryptophan metabolism enhances interferon therapy in kidney cancer

**DOI:** 10.18632/oncotarget.11658

**Published:** 2016-08-27

**Authors:** Josephine F. Trott, Jeffrey Kim, Omran Abu Aboud, Hiromi Wettersten, Benjamin Stewart, Grace Berryhill, Francisco Uzal, Russell C. Hovey, Ching-Hsien Chen, Katie Anderson, Ashley Graef, Aaron L Sarver, Jaime F. Modiano, Robert H. Weiss

**Affiliations:** ^1^ School of Medicine, Division of Nephrology, University of California Davis, Davis, CA, USA; ^2^ Department of Animal Science, University of California Davis, Davis, CA, USA; ^3^ Comprehensive Cancer Center, University of California Davis, Davis, CA, USA; ^4^ Biosciences and Biotechnology Division, Lawrence Livermore National Laboratory, Livermore, CA, USA; ^5^ California Animal Health and Food Safety Lab, School of Veterinary Medicine, University of California, Davis, San Bernardino, CA, USA; ^6^ Animal Cancer Care and Research Program, College of Veterinary Medicine, University of Minnesota, St Paul, MN, USA; ^7^ Department of Veterinary Clinical Sciences, College of Veterinary Medicine, University of Minnesota, St Paul, MN, USA; ^8^ Masonic Cancer Center, Minneapolis, MN, USA; ^9^ Center for Immunology, Minneapolis, MN, USA; ^10^ Stem Cell Institute University of Minnesota, Minneapolis, MN, USA; ^11^ Medical Service, VA Northern California Health Care System, Sacramento, CA, USA, USA; ^12^ Sanford Consortium for Regenerative Medicine, UC San Diego, La Jolla, CA, USA

**Keywords:** tryptophan, kynurenine, renal cell carcinoma, interferon-alpha, indolamine-2, 3-dioxygenase

## Abstract

Renal cell carcinoma (RCC) is increasing in incidence, and a complete cure remains elusive. While immune-checkpoint antibodies are promising, interferon-based immunotherapy has been disappointing. Tryptophan metabolism, which produces immunosuppressive metabolites, is enhanced in RCC. Here we show indolamine-2,3-dioxygenase-1 (IDO1) expression, a kynurenine pathway enzyme, is increased not only in tumor cells but also in the microenvironment of human RCC compared to normal kidney tissues. Neither kynurenine metabolites nor IDO inhibitors affected the survival or proliferation of human RCC or murine renal cell adenocarcinoma (RENCA) cells *in vitro*. However, interferon-gamma (IFNγ) induced high levels of IDO1 in both RCC and RENCA cells, concomitant with enhanced kynurenine levels in conditioned media. Induction of IDO1 by IFNα was weaker than by IFNγ. Neither the IDO1 inhibitor methyl-thiohydantoin-DL-tryptophan (MTH-trp) nor IFNα alone inhibited RENCA tumor growth, however the combination of MTH-trp and IFNα reduced tumor growth compared to IFNα. Thus, the failure of IFNα therapy for human RCC is likely due to its inability to overcome the immunosuppressive environment created by increased IDO1. Based on our data, and given that IDO inhibitors are already in clinical trials for other malignancies, IFNα therapy with an IDO inhibitor should be revisited for RCC.

## INTRODUCTION

Kidney cancer is one of the few malignancies that show an increasing incidence in the United States, possibly due to the prevalence of obesity and the metabolic syndrome in the Western world [1, 2]. The treatment of metastatic RCC has evolved from the early, and relatively ineffective, use of single-agent immunomodulatory agents such as interferon-alpha (IFNα) and interleukin-2 (IL-2) [3, 4], to the use of targeted therapeutics such as the multi-kinase and mTOR inhibitors as well as the checkpoint inhibitors. Nevertheless, the continued lack of therapeutic targets that produce a sustained remission for this disease has led our group and others to search for novel and heretofore untested approaches based on its metabolic features.

In mammalian tissues, the essential amino acid tryptophan can be metabolized to serotonin, melatonin, kynurenic acid, picolinic acid, and NAD+, the latter three compounds being produced *via* the kynurenine pathway, which is the major pathway for tryptophan metabolism [5, 6]. The kynurenine pathway of tryptophan metabolism influences the innate and adaptive immune systems through production of the metabolites kynurenine, 3-hydroxy kynurenine (3-HK), 3-hydroxyanthranilic acid (3-HAA) and quinolinate *via* IFNγ-induced indolamine-2,3-dioxygenase-1 (IDO1) activity [6]. The known effects of kynurenine metabolites include inhibition of T-cell proliferation, downregulation of NK cell responses and activation of T regulatory (Treg) cells [6]. By combining non-targeted metabolomic and proteomic analysis of human RCC tissue, we have recently uncovered striking upregulation of the kynurenine pathway of tryptophan metabolism in clear cell RCC (ccRCC) [7], confirming our metabolomics data from urine analysis in ccRCC patients [8]. These data suggest that one of the most proximal enzymes in the kynurenine pathway is likely up-regulated in ccRCC. Concomitantly and conspicuously, the other branches of tryptophan metabolism (for example serotonin and indole-3-acetate) are markedly downregulated in RCC [7].

The most proximal enzyme in the kynurenine pathway that is expressed in tumors and immune cells is IDO1 [9], while its paralog IDO2 is expressed in dendritic cells. These two enzymes act *via* multiple mechanisms to enable tumors to evade critical immune surveillance [10, 11]. In light of these associations and our previous data, we asked whether inhibition of the kynurenine pathway *in vivo* inhibits tumor growth in a mouse model of immune-stimulated kidney cancer. We now show that growth of several human RCC cell lines as well as RENCA cells incubated in the presence of IDO inhibitors, kynurenine, or its metabolites, is not altered *in vitro*, yet growth of RENCA tumors in immune-competent mice *in vivo* is attenuated when IFNα is administered concurrently with the competitive IDO1 and IDO2 inhibitor methyl-thiohydantoin-DL-tryptophan (MTH-trp). Our results suggest that the mechanism of this effect is associated with the production of IDO1 by endothelial, tumor and CD68+ immune cells leading to tryptophan catabolism into kynurenine, which can be inhibited by MTH-trp. In light of our data, it is logical to revisit IFN-based immunotherapy for RCC but in future trials it should be combined with IDO1 inhibitors.

## RESULTS

### IDO1 expression is increased in RCC compared to adjacent normal tissue

**Figure 1:**
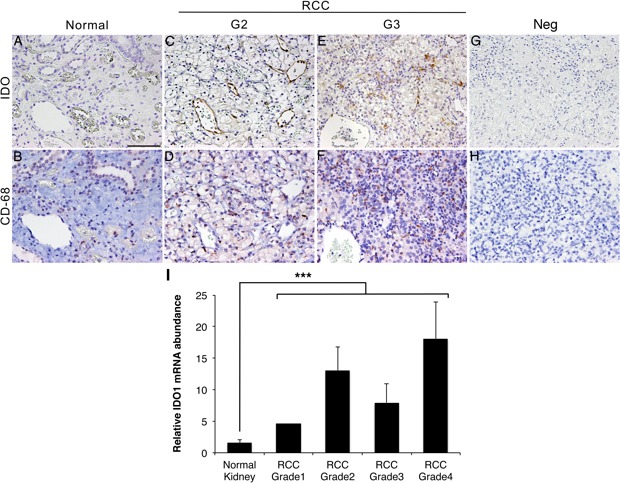
IDO1 is upregulated in human RCC **A.**, **C.**, **E.**, **G.** Immunohistochemistry with IDO1 antibody of normal human kidney and RCC (grades 2 and 3). **A**. Adjacent normal kidney **C.** Grade 2 RCC **E.** Grade 3 RCC **G.** Grade 2 RCC without IDO1 antibody. **B., D., F., H.** Immunohistochemistry with CD68 antibody in serial sections to A, C, E and G were stained for CD68. **B**. Adjacent normal kidney **D.** Grade 2 RCC **F.** Grade 3 RCC **H.** Grade 2 RCC without CD68 antibody. **I**. Expression of IDO1 mRNA measured by qPCR in grades 1-4 of RCC and adjacent normal kidney tissues. ***P < 0.0001. Scale bar = 100 μm.

Of the several enzymes that convert tryptophan to kynurenine, IDO1 is widely expressed across tissues whereas TDO2 is expressed primarily in the liver [12]. We therefore examined the distribution of IDO1 in ccRCC tumors of grades 2, 3 and 4, as well as in adjacent normal tissue byimmunohistochemistry, and we measured IDO1 mRNA levels by qRT-PCR. RCC tissues showed upregulated IDO1 mRNA expression compared with adjacent normal kidneys (Figure 1). IDO1 staining localized to endothelial cells, tumor cells and inflammatory cells resembling tissue macrophages or myeloid cells in grades 2 and 3 RCC (Table 1; Figure 1; raw data in Table 2). Large areas of necrosis in all grade 4 RCC samples precluded an accurate assessment of staining in these tissues (data not shown). The staining patterns were distinct in grades 2 *vs*. 3: cells in microvessels within the parenchyma accounting for a greater proportion of the staining in grade 3 tumors and cells lining small venules accounting for most of the staining in grade 2 tumors (Figure 1). The apparent IDO staining of macrophages was assessed by staining serial sections of RCC for CD68, an antigen expressed primarily by macrophages/monocytes and dendritic cells as well as by some neutrophils and large lymphocytes. Most CD68-positive cells were interstitial and more abundant in grades 2 and 3 compared to normal (Figure 1; Table 1). Some CD68-positive cells were intimately associated with blood vessels, including capillaries. Indeed, there were more CD68-positive interstitial cells than IDO1-positive interstitial cells in RCC tissues (Table 1), but virtually all the IDO1-positive interstitial cells localized to CD68+ areas (Figure 1), suggesting that IDO1 is present in inflammatory cells as well as in tumor and endothelial cells.

**Table 1:** Immunohistochemistry staining score of renal cell carcinoma (RCC) and adjacent normal kidney tissue

Antibody	IDO1^1^	IDO1	IDO1	CD68^2^
Tissue	Endothelial cells	Neoplastic cells	Interstitial cells	Interstitial cells
Kidney	0±0	0±0	0±0	0.67±0.28
Grade 2^3^	2.00±1.00	0.67±0.58	1.00±0	2.50±0.50
Grade 3	2.33±0.58	0±0	1.00±0	3.83±1.26

1 Score for indolamine-2,3-dioxygenase 1 (IDO1): 0=no staining, 1=1-10%, 2=10-20%, 3=20-40% staining, respectively

2 Score for CD68: 0=no staining, 1=1-10%, 2=10-20%, 3=20-30%, 4=30-40%, 5=40-50% positive interstitial cells

3 Grade of RCC

**Table 2:** Immunohistochemistry staining score of RCC and adjacent normal kidney tissue

Antibody	IDO1^3^	IDO1	IDO1	CD68^4^
Tissue	Endothelial cells	Neoplastic cells	Interstitial cells	Interstitial cells
Kidney Neg^1^	0	0	0	n/a
Kidney-1	0	0	0	0.5
Kidney-2	0	0	0	0.5
Kidney-3	0	0	0	1
Grade 2^2^ Neg	0	0	0	0
Grade 2-1	1	0	1	2.5
Grade 2-2	3	1	1	3
Grade 2-3	2	1	1	2
Grade 3 Neg	0	0	0	0
Grade 3-1	2	0	1	4
Grade 3-2	2	0	1	5
Grade 3-3	3	0	1	2.5

1 Neg=secondary antibody only

2 Grade of renal cell carcinoma (RCC)

3 Score for IDO1: 0=no staining, 1=1-10%, 2=10-20%, 3=20-40% staining, respectively

4 Score for CD68: 0=no staining, 1=1-10%, 2=10-20%, 3=20-30%, 4=30-40%, 5=40-50% positive interstitial cells

### Kynurenine pathway metabolites and IDO inhibitors have minimal effects on RCC viability *in vitro*

**Figure 2:**
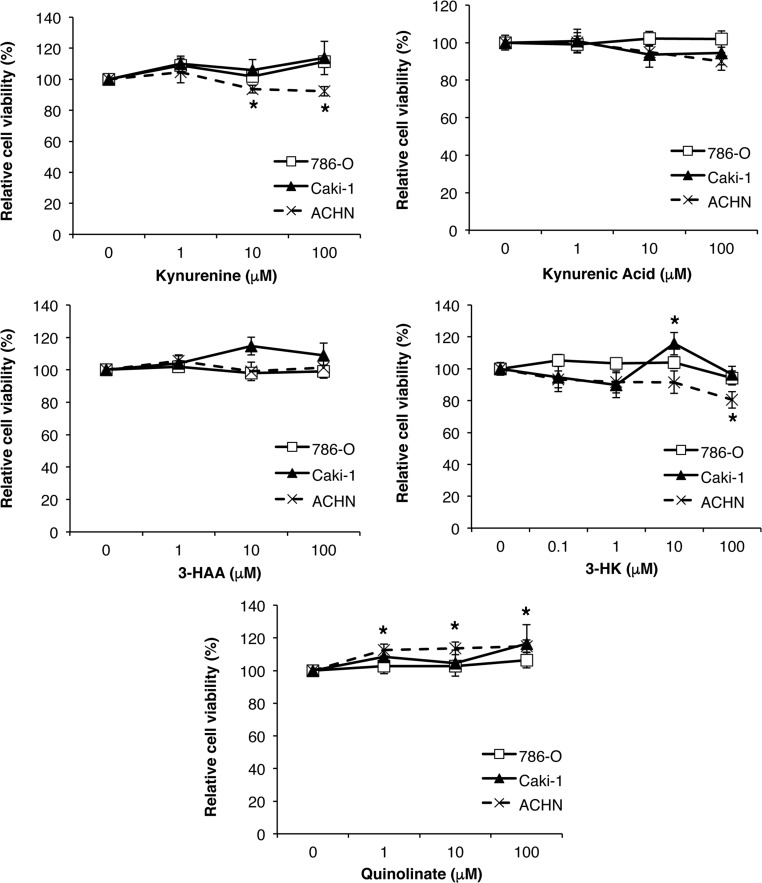
Minimal effect of tryptophan metabolites 3-hydroxyanthranilic acid (3-HAA), 3-hydroxy-DL-kynurenine (3-HK), kynurenine, quinolinate and kynurenic acid on viability of human RCC cell lines Equal numbers of cells were plated in 96-well plates, treated (*n* = 6 wells/treatment) for three days with different doses of each metabolite then cell viability measured using an MTT assay. Data are an average of two independent experiments and representative of at least three experiments. *P ≤ 0.05.

**Figure 3:**
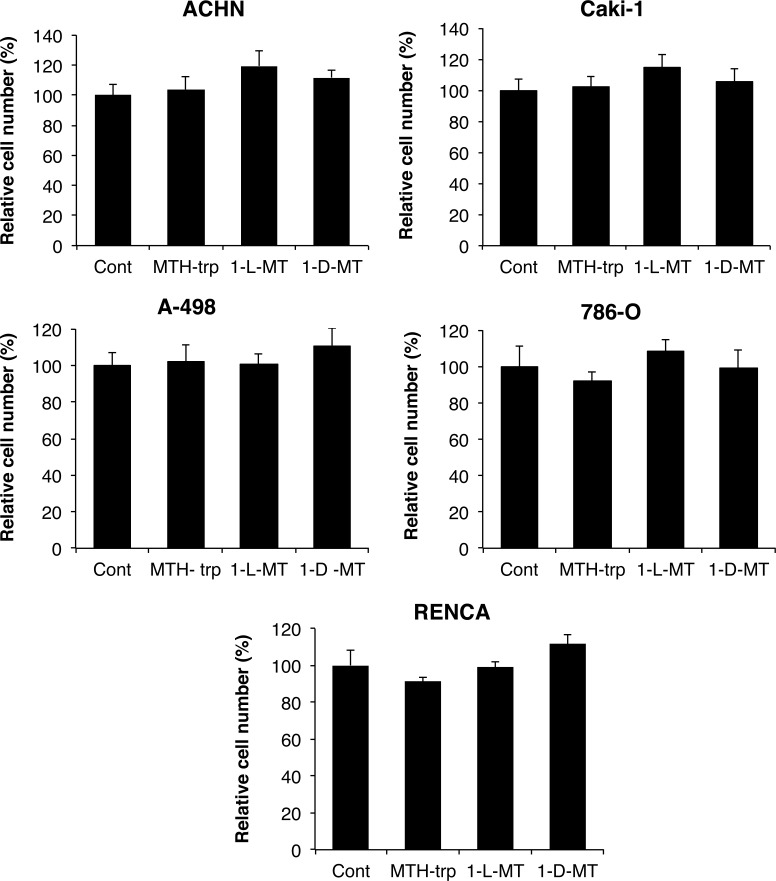
Minimal effect of the IDO inhibitors methyl-thiohydantoin-DL-tryptophan (MTH-trp), 1-methyl-L-tryptophan (1-L-MT) and 1-methyl-D-tryptophan (1-D-MT) on human RCC and RENCA cell growth Equal numbers of cells were plated in 96-well plates, treated (*n* = 6 wells/treatment) for three days with different IDO inhibitors or 0.1% DMSO, then final cell numbers measured using the methylene blue assay. Data are representative of three independent experiments.

In light of our earlier finding that kynurenine pathway metabolites are upregulated in RCC [7] and based on previous reports demonstrating toxicity of these metabolites for murine and human T-cells [6, 17, 18], we first evaluated the direct effects of the kynurenine pathway metabolites 3-HAA, 3-HK, kynurenine, quinolinate and kynurenic acid on the viability of the three human RCC cell lines 786-O, ACHN and Caki-1. There were minor effects of just three metabolites, but only on ACHN cell viability (Figure 2). In addition, the IDO1 and/or IDO2 competitive inhibitors 1-L-MT, 1-D-MT and MTH-trp had no effects on cell growth across human and mouse renal cancer cell lines (Figure 3). Thus, neither tryptophan metabolites nor IDO inhibitors affect the viability or proliferation of RCC cells *in vitro.*

### IDO1 expression in RCC cell lines is stimulated by interferons

**Figure 4:**
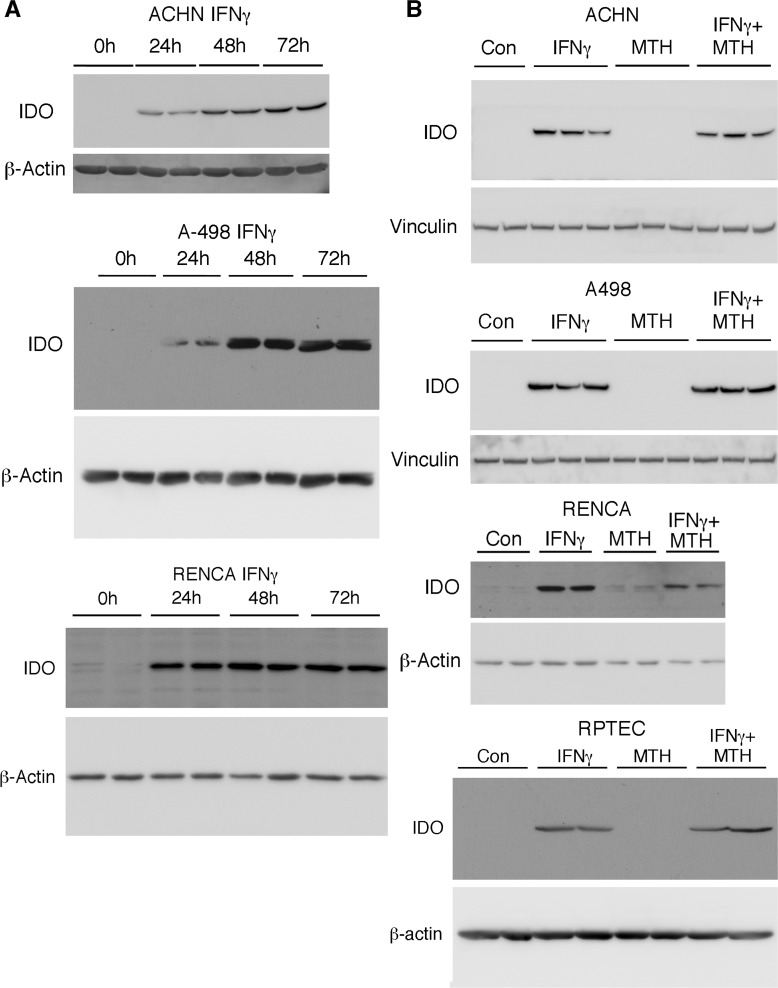
IFNγ induces IDO1 in RCC and normal renal proximal tubular epithelial cells, and levels are unaffected by IDO inhibition **A.** Human RCC cells (A-498; ACHN; 50 ng/ml hIFNγ) or RENCA cells (100 ng/ml mIFNγ) were incubated with IFNγ for the times indicated, followed by immunolotting for IDO1. **B.** Human RCC cells, RENCA cells, or normal human RPTEC were incubated with IFNγ in the presence or absence of MTH-trp for 72 h followed by immunoblotting for IDO1. Data shown are representative of at least two independent experiments.

**Figure 5:**
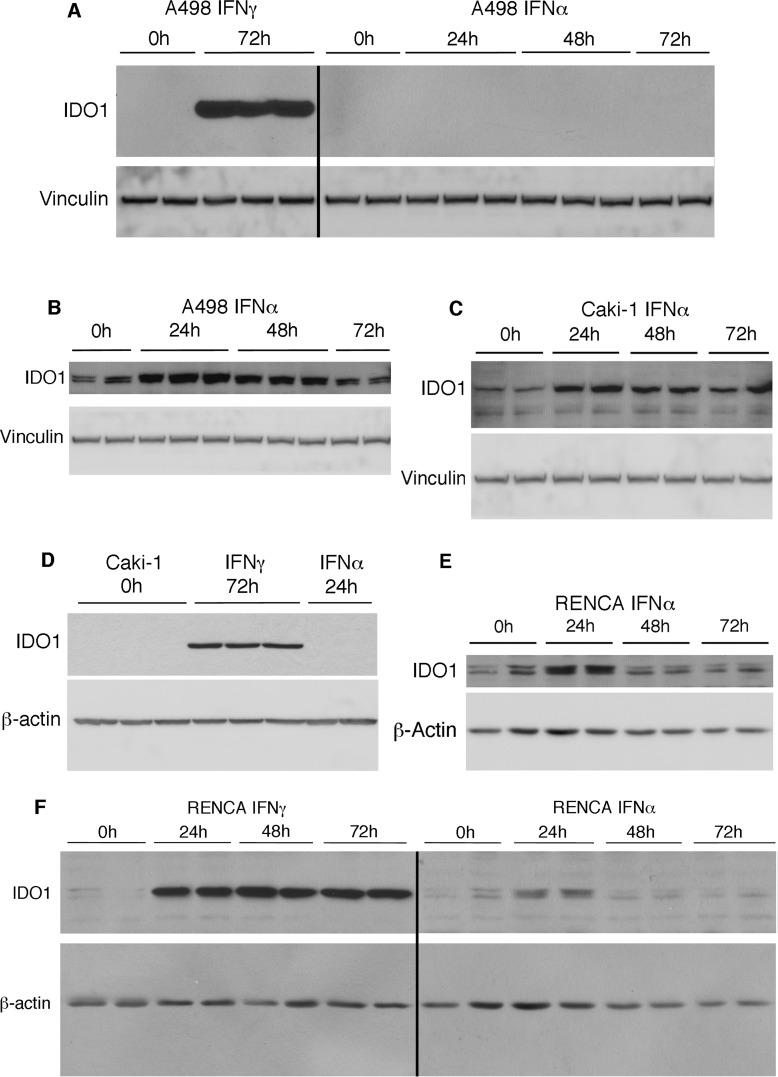
IFNα induces IDO1 in human RCC and RENCA cells The indicated RCC cells were incubated with IFNα or IFNγ for the times indicated and immunoblotted for IDO1 and vinculin or β-actin. **A.** A498 cells stimulated with either IFNγ for 72h or IFNα for 24-72h. Chemiluminescent exposure times were the same for all samples. **B.** A498 cells stimulated with IFNα for 24-72h from **A**. after a longer exposure. **C.** Caki-1 cells stimulated with IFNα for 24-72h. **D.** Caki-1 cells stimulated with either IFNγ for 72h or IFNα for 24h. **E.** RENCA cells stimulated with IFNα for 24-72h. **F.** RENCA cells stimulated with IFNγ from Figure 4A and RENCA cells stimulated with IFNα from Figure 5E exposed for the same length of time. Data shown are representative of two independent experiments.

The transcription of IDO1 mRNA, which is mainly expressed in RCC rather than normal kidney (Figure 1), has been shown to be induced by IFNγ [19]. Because early (and still extant) chemotherapies for advanced RCC include IFNα [3, 20], we examined the temporal induction of IDO1 by both IFNγ and IFNα in human (ACHN and A498) and mouse RCC (RENCA) cells. Levels of IDO1 were markedly increased in RENCA cells by mouse IFNγ (100 ng/ml), and in two human RCC cell lines by human IFNγ (50 ng/ml), with maximal induction after 48-72h (Figure 4A). As expected, there was no effect of the IDO1/2 inhibitor MTH-trp on levels of IFNγ-stimulated IDO1 protein (Figure 4B). The expression of IDO1 was also induced by IFNα in human RCC and RENCA cells *in vitro,* but maximal induction occurred after 24h (Figure 5), which was earlier than the IDO1 induction by IFNγ (Figure 4). In addition, the relative magnitude of IDO1 protein induction by IFNα was considerably less than that initiated by IFNγ (Figure 5). A similar phenomenon of differential IFN regulation of downstream effectors has been reported in primary human fibroblasts [21].

### Kynurenine pathway metabolism *in vitro* is increased by IFNγ and attenuated by IDO inhibition

**Figure 6:**
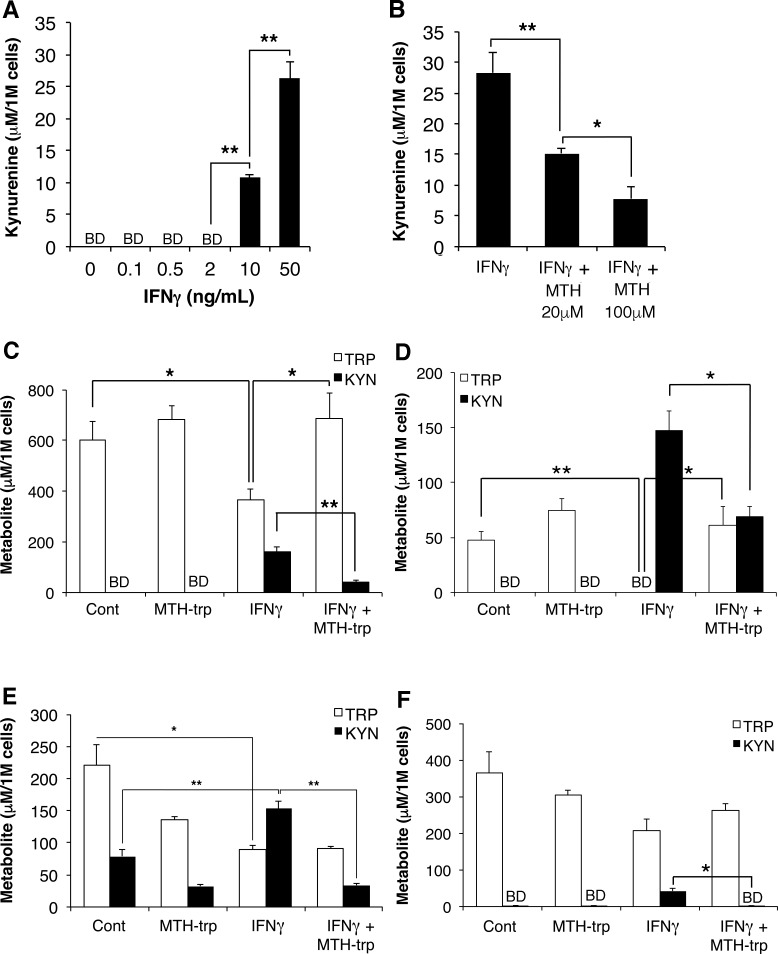
The IFNγ-induced increase in tryptophan catabolism is inhibited by MTH-trp Cells were treated as indicated for 72 h and conditioned media subjected to HPLC to measure tryptophan (TRP) and kynurenine (KYN) levels using authentic standards. **A.** Dose curve for IFNγ (0-50 ng/ml) added to 786-O cells (*n* = 2) alone, or **B.** IFNγ (50 ng/ml) in combination with MTH-trp (0-100 μM). Media was collected from **C.** ACHN, **D.** A-498, **E.** RENCA and **F.** RPTEC cells (*n* = 3) treated with either human IFNγ (50 ng/ml) or mouse IFNγ (100 ng/ml) with or without MTH-trp (100 μM). Control cells (Con) were treated with 0.1% DMSO only. Results are representative of two independent experiments and expressed as concentration in media per 1 × 10^6^ (1M) cells. **P < 0.01; *P < 0.05. BD: below limits of detection (5 μM).

To determine whether inhibition of IDO attenuates the kynurenine pathway in RCC cells *in vitro,* and as a prelude to *in vivo* studies, we evaluated the dose-response of the kynurenine pathway to IFNγ which, of the two IFNs, evoked the higher induction of IDO1. Incubation of 786-O cells with IFNγ stimulated maximal production of kynurenine (Figure 6A). We subsequently evaluated the ability of the commonly-used specific IDO1 inhibitor 1-L-MT [14] as well as MTH-trp, which is a competitive inhibitor of IDO1 and IDO2 [22, 23], to inhibit the activity of IFNγ-induced IDO1. While treatment with 1-L-MT (100 μM) did not reduce the IFNγ-induced production of kynurenine (data not shown), an MTH-trp dose curve revealed that 100 μM MTH-trp (25.9 μg/ml) realized maximal inhibition of kynurenine production (Figure 6B). We then evaluated this process in several RCC cell lines and RPTEC cells. Treatment with IFNγ induced kynurenine secretion and significantly reduced tryptophan concentrations in the media from ACHN, A-498 and RENCA cells (Figure 6C-6E), while IFNγ induced kynurenine secretion by RPTEC cells was minimal, with no effect on tryptophan concentrations (Figure 6F). Notably the combination of MTH-trp and IFNγ significantly inhibited kynurenine production in all cell lines compared to IFNγ treatment (Figure 6C-6F). Thus the kynurenine pathway is activated in RCC cells in response to IFNγ and can be inhibited by the dual IDO1 and IDO2 inhibitor MTH-trp.

### Tumor growth *in vivo* is inhibited by MTH-trp and IFNα

**Figure 7:**
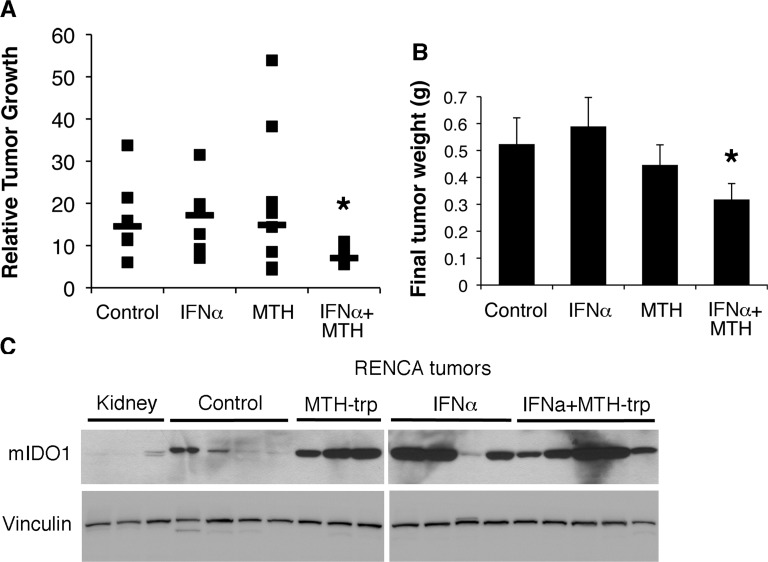
The IDO1 inhibitor MTH-trp enhances the anti-tumor effect of IFNα *in vivo* Male Balb/cJ mice (*n*= 37) were injected subcutaneously with 500,000 RENCA cells on day 0. Treatments were initiated on day 7 and mice were euthanized on day 21; MTH-trp (4 mg/mouse) was injected daily (IP); IFNα (25,000U) was injected 5 d/week (SC). **A.** Resected tumors were measured with calipers and the relative tumor growth rate for each mouse is plotted as size on day 21/size on day 7. Median data points are indicated with a horizontal line. *P < 0.07 compared to Con (control) and MTH. *P < 0.05 compared to IFNα. **B.** Final weights of resected RENCA tumors. *P < 0.05 compared to IFNα. *P < 0.1 compared to Con. Data are least squares means ± standard error. **C**. Proteins extracted from Balb/c mouse kidneys or RENCA tumors from control mice or mice treated with MTH-trp, IFNα or IFNα+MTH-trp were probed for IDO1 expression by immunoblotting.

Given that (i) IDO1 expression is elevated in RCC (Figure 1), (ii) kynurenine pathway metabolites are increased in RCC [7], and (iii) the immunomodulatory agent IFNα can induce IDO1 expression in RCC cells (Figure 5), we conducted an *in vivo* study using RENCA cells in syngeneic Balb/c mice treated with IFNα and MTH-trp. IFNα rather than IFNγ was utilized in these experiments as it has previously been used to treat RCC in the clinic. While neither MTH-trp nor IFNα alone inhibited tumor growth, the combination of IFNα and MTH-trp significantly reduced both the rate of tumor growth and the final tumor weight compared to treatment with IFNα alone (*P* < 0.05; Figure 7A and 7B). The combination of IFNα and MTH-trp also reduced the tumor growth rate compared to that in control animals (*P* = 0.07; Figure 7A) and the MTH-trp treated animals (*P* = 0.07; Figure 7A) although this did not reach the *P* < 0.05 threshold. At necropsy, IDO1 protein expression was higher in nearly all of the RENCA tumors compared to that in three normal kidneys (Figure 7B), similar to the elevated expression of IDO1 in human RCC (see Figure 1).

### MTH-trp does not alter the topology or composition of the intratumoral immune microenvironment *in vivo*

Previous work has shown that inhibition of IDO-1 restores activity of T cells in the tumor environment [24], but the potential of IDO inhibition to alter the overall tumor immune landscape has not been determined. We therefore performed a second *in vivo* study using the heterotopic RENCA model where animals were treated with vehicle or with MTH-trp alone. After 10 d of treatment, the tumors were harvested, a section from each was fixed for routine pathological analysis, and the rest was dissociated into a suspension of single cells to interrogate their stromal, immune and inflammatory composition by multi-parameter flow cytometry. The tumors were extremely anaplastic with marked anisocytosis and anisokaryosis, frequent mitoses, bizarre giant cells/syncytia, and marked invasion into surrounding subcutaneous tissues. The larger tumors had extensive areas of necrosis with secondary neutrophilic and histiocytic inflammation. The subcutaneous tissues surrounding the tumors contained mild to moderate numbers of macrophages with fewer lymphocytes and plasma cells. Among the subsets analyzed by flow cytometry, there was substantial variation in the number of tumor-infiltrating leukocytes. Neither the total number of intratumoral immune cells nor the relative number of granulocytes (GR1^+^, CD11b^+^, or GR-1/CD11b^+^), B cells (CD20+), T cells (CD3^+^), dendritic cells (CD11c^+^), NK cells (NK1.1^+^), NK-T cells (NK1.1^+^/ CD3^+^), or fibroblast and endothelial stromal cells (PDGFRα^+^) in the tumors was changed by MTH-trp treatment.

### TDO2 is not elevated in RCC cells and tissues

**Figure 8:**
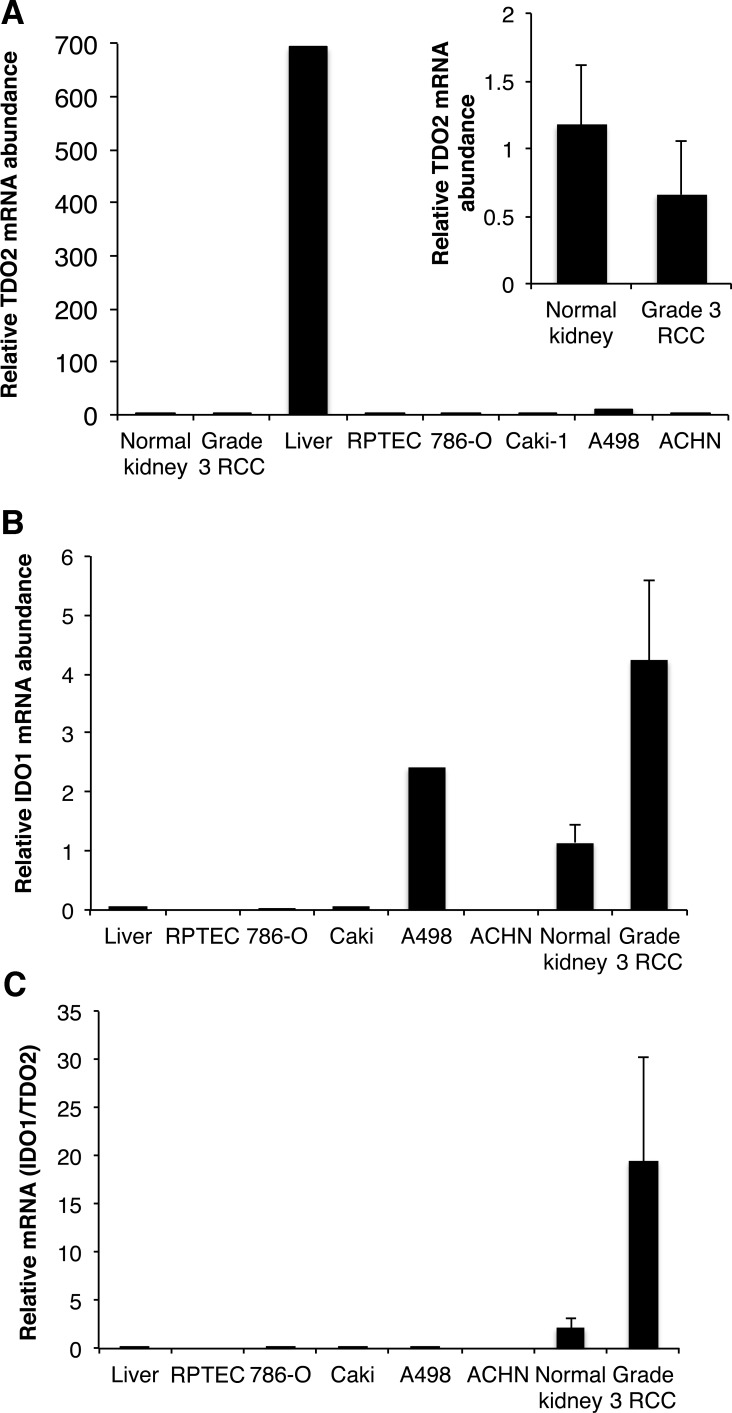
Indolamine-2,3-dioxygenase 1 (IDO1) is expressed at higher levels than tryptophan-2,3-dioxygenase *(TDO2)* in kidney and RCC tissues RNA was reverse transcribed and subjected to qPCR for *IDO1* and *TDO2* mRNA and corrected for *PPIA* and *RPS13* mRNA levels. **A.** Human liver (*n* = 1), grade 3 RCC (*n* = 5) and matched normal kidney (*n* = 5), RPTEC (n=1), A-498 (*n* = 1), 786-O (*n* = 1), ACHN (*n* = 1) and Caki-1 (*n* = 1). **B.** *IDO1* mRNA quantity in the same samples analyzed in **A**. **C.** Ratio of *IDO1/TDO2* mRNA in the same samples analyzed in **A**.

**Figure 9:**
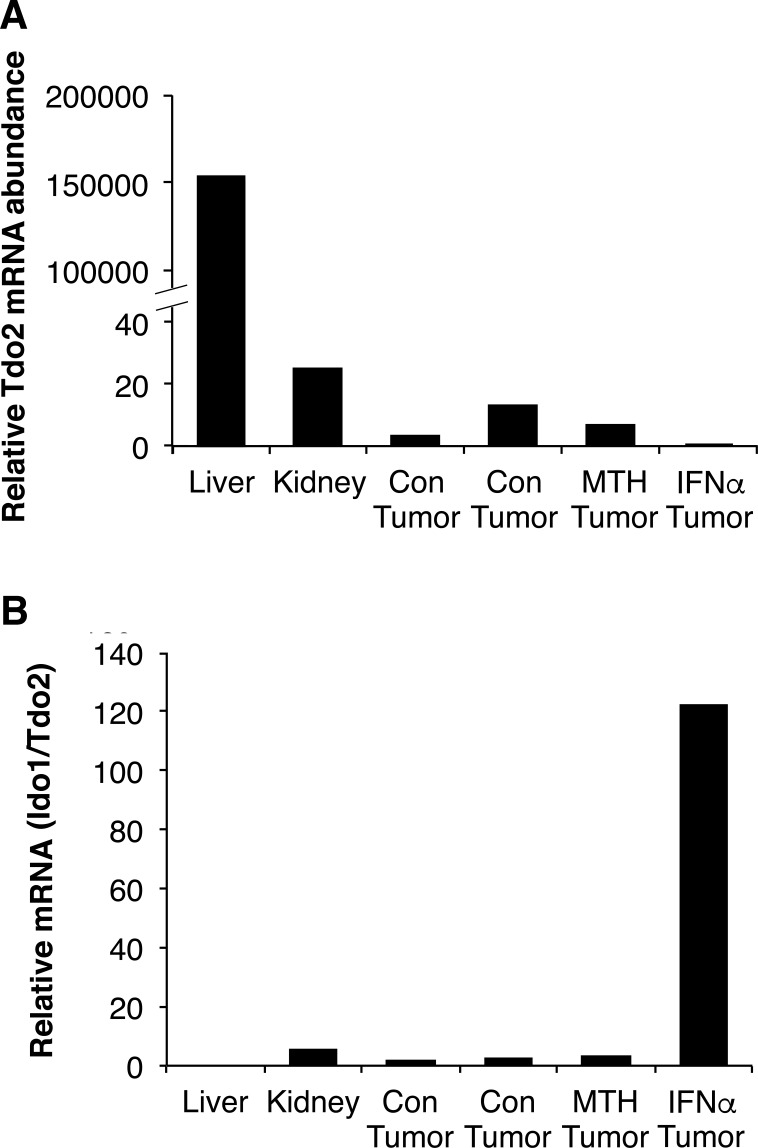
Mouse IDO1 expression levels are much higher than TDO2 in kidney and RENCA tumors Mouse *Tdo2* expression was corrected for *Rn18S* and *Gapdh* mRNA levels. Mouse liver (*n* = 1), mouse kidney (*n* = 1) and RENCA tumors (*n* = 4) from control (Con), MTH-trp (MTH) or IFNα treated mice.

Because IDO1, IDO2 and TDO2 all catabolize tryptophan to kynurenine, any of these enzymes could activate the kynurenine pathway in RCC, a possibility we evaluated by qRT-PCR. Expression of *IDO2* mRNA was detected in human and mouse kidney, but not in grade 3 RCC or RENCA tumors (data not shown). As expected, we found that *TDO2* mRNA was most abundant in both human and mouse liver (Figures 8A and 9A). There was no difference in h*TDO2* mRNA expression between normal kidney and grade 3 RCC tissues (*P* = 0.5; Figure 8A). However, h*IDO1* mRNA was expressed at higher levels than *TDO2* in normal kidney and was elevated further in grade 3 RCC (Figures 8B and 8C). Similarly, m*Ido1* mRNA was expressed at higher levels than m*Tdo2*in normal kidney and RENCA tumors (Figure 9B). Combined with our data showing elevated IDO1 protein and mRNA expression in grades 2, 3 and 4 RCC compared to normal kidneys (see Figure 1), we conclude that TDO2 and IDO2 are unlikely to be involved in the grade-dependent elevation of the kynurenine pathway that occurs in RCC.

## DISCUSSION

Long before the recent discovery and therapeutic use of antibodies against immune checkpoints, patients with RCC were treated with the immunomodulatory agents IFNα and IL-2 [3, 4]. In addition to the disappointing responses of these cancers to such treatments, patients undergoing these therapies experienced serious adverse effects that, with the advent of targeted therapies like mTOR and VEGFR inhibitors, led to the near-abandonment of IFNα and IL-2 as mainstream RCC therapies [20, 25, 26]. However, the partial success of immunomodulatory agents, as well as several recent successful clinical trials using antibodies against immune checkpoints, has underscored the immunogenicity of RCC [27] such that the use of interferons should be revisited. Our data from the current study support the notion that at least one reason for the common failure of IFNα therapy in treatment of human disease is due to its further induction of IDO1, which is already upregulated in RCC as evidenced by such high levels of immunosuppressive kynurenine pathway metabolites that they appear in the urine of RCC patients [8]. On the other hand, the novel idea of using the combination of IFNα and an IDO1 inhibitor results in salutary effects on tumor growth in mice: in this situation the immune stimulatory properties of IFNα are left intact while the immunosuppressive activities (through the generation of kynurenine and its metabolites) are blocked. Whether this will hold true in human disease is a question which should be addressed in future clinical trials.

Earlier non-targeted metabolomics studies in our laboratory showed increased levels of kynurenine pathway metabolites in the urine of ccRCC patients [8], as well as in human RCC xenografts [28] and RCC tissues [7]. Those findings served as the basis for the current study in which we evaluated whether the kynurenine pathway was operative and could be therapeutically targeted in RCC. The enzymes most proximal to tryptophan, which result in activation of this pathway, are IDO1, IDO2 and TDO2, and all of these enzymes have been shown to be upregulated in a variety of cancers [10, 29]. Upregulation of IDO can inhibit T-cell activation through a combination of tryptophan starvation as well as via the action of kynurenine and its metabolites 3-HK, 3-HAA and quinolinate [11, 30, 31]. Plasmacytoid dendritic cells expressing IDO can also activate mature Treg cells and convert naïve T-cells into Treg cells [11, 30], where IDO downregulates production of IFNα by dendritic cells [11]. Our data suggest that MTH-trp reduces IDO activity and production of kynurenine pathway metabolites and thus potentially prevents or reverses these effects. Moreover, we also demonstrate that there is substantial IDO1 but relatively little TDO2, in human RCC tissue and RENCA tumors, suggesting that the TDO2-driven immune suppression mechanism is less relevant to RCC.

Tumor endothelial cells appear to express IDO1 in human RCC as has been previously suggested [13]. We also find that IDO1 was produced by tumor cells and by tumor-associated interstitial cells that may be CD68+ macrophages or may be other “round” cells observed in these sections, including non-macrophage myeloid derived cells. Our finding that IDO1 immunoreactivity was confined to tumor blood vessels (and not in adjacent normal kidney vessels), suggests that RCC tumors are using the well-described immunosuppressive mechanism of intra-tumoral activation of IDO1 expression and enhancement of the local kynurenine pathway [6]. Consistent with these data, kynurenine and its metabolites individually had no effect on RCC cell growth *in vitro* while many of these compounds are toxic to immune cells [6].

Given what is known about alterations in the immune environment which occur in response to tryptophan catabolism [32], we expected that the major effects of MTH-trp would manifest as an alteration in the tumor's immune environment; however our data (at least after 10 days) do not support that hypothesis as treatment of the animals with MTH-trp did not alter the anatomical, topological, or phenotypic composition, nor the absolute number tumor-infiltrating immune cell subsets. Thus it is likely that tryptophan metabolites, or the administration of IFNα and an IDO1 inhibitor, are in the short term causing their observed effects upon tumor growth by indirect actions upon immune cells, such as altered functionality of intratumoral leukocytes. Further and longer-term studies are required to fully evaluate the effects of MTH-trp on the functionality of these subsets, particularly effector T cells in renal cell carcinoma in general and in the RENCA model in particular.

There are several ongoing clinical studies using IDO inhibitors, either singly or in combination, in refractory RCC. Two combination IDO inhibitor and checkpoint antibody studies (clinicaltrials.gov NCT02178722, NCT02318277), a combination IDO inhibitor and JAK inhibitor study in advanced solid tumors (NCT02559492), and an IDO inhibitor alone study on refractory solid tumors (NCT02048709) are all recruiting. However, none of these studies are complete and none have utilized interferon in combination with an IDO inhibitor; thus our pre-clinical study is unique and novel.

In summary, we have extended our previous findings showing reprogramming of tryptophan metabolism in RCC leading to production of immunosuppressive metabolites. In this study, we have demonstrated that pharmacologic attenuation of this pathway in the presence of immunomodulatory IFNα decreases tumor growth in the RENCA mouse model of kidney cancer. Despite the absence of an effect on the composition of the tumor immune microenvironment in a subacute setting, MTH-trp may alter the functionality of intratumoral leukocytes, setting the stage for improved therapeutic effects of IFNα. Due to the current clinical availability and FDA approval of both compounds, and the established immunogenicity of RCC, this work could readily and easily be translated into the clinic.

## MATERIALS AND METHODS

### Materials

Recombinant mouse IFNα-2 (eBiosciences, San Diego, CA), recombinant human IFNα-2a (Gemini Bio-Products, Sacramento, CA), recombinant mouse IFNγ (PeproTech, Rocky Hill, NJ) and recombinant human IFNγ (PeproTech) were purchased as described. Methyl-thiohydantoin-DL-tryptophan (MTH-trp) was from Cayman Chemical (Ann Arbor, MI) and dissolved in DMSO. L-tryptophan, kynurenine, 3-HK, kynurenic acid, 3-HAA, quinolinate, 1-methyl-L-tryptophan (1-L-MT) and 1-methyl-D-tryptophan (1-D-MT) were all from Sigma-Aldrich (St Louis, MO).

### *In vivo* tumor studies

Animal experiments were performed in accordance with guidelines set forth by the Institutional Animal Care and Use Committee at UC Davis. Seven-week old male Balb/cJ mice (Jackson Laboratories, Bar Harbor, ME) had *ad libitum* access to food and water. For the tumor growth study, mice were injected with 5 × 10^5^ RENCA cells from passage 7 to 11 (100 μl cells in OptiMEM with 33% BD Matrigel Matrix (Corning, Tewksbury, MA) SC in the right flank). For the flow cytometry endpoint, cells were passage 5. Seven days later, treatments were initiated and tumors measured daily with calipers. Mice were injected 5 d/week with murine IFNα 2 (25,000 U/mouse, SC) or saline, and daily with MTH-trp (4 mg/mouse, IP) or DMSO. They were euthanized after being treated for 10 d (flow cytometry) or 14 d (tumor burden and therapeutic efficacy). Resected tumors were measured, weighed, and volume assessed by V = 4/3*3.142*(W/2)*(L/2)*(D/2). Tumor pieces were frozen or fixed in 10% formalin.

### Isolation of tumor-infiltrating cells and flow cytometry

Tumors and spleens were homogenized into a suspension of single cells then stained with a panel of fluorochrome-conjugated antibodies to evaluate T cells (CD3^+^), B cells (CD19^+^), dendritic cells (CD11c^+^), granulocytes (Gr1^+^), monocytes (CD11b^+^), stromal cells (PDGFRα^+^), NK cells (NK1.1^+^) and NKT cells (CD3^+^/NK1.1^+^). CD4, CD8, Th17, and Treg T cell subsets, myeloid derived granulocytic and monocytic suppressor cell subsets, and T-cell activation status (naïve, conventional effector, effector memory, and central memory) were further characterized according to expression of CD25, CD69, CD62L, and CD44 on the cell surface and intracellular Foxp3, RORγT, IL-2, IL-10, IL-17, and IFNγ as previously described [33]. Stained cells were analyzed using a BD LSRII flow cytometer (BD Biosciences, San Jose, CA), and data processed using Flowjo software (Treestar, Ashland, OR).

### Cell culture

All cells were confirmed mycoplasma-free. Mouse RENCA cells (Murphy, 1973 #73) were a gift from Dr. Montjazeb (Dept of Radiation Oncology, University of California, Davis) and were verified by PCR to contain mouse and not human DNA (data not shown). RENCA cells were cultured in RPMI-1640 with 4 mM glutamine, 10% FBS, 1x MEM non-essential amino acids, 1 mM sodium pyruvate, 15 mM HEPES, penicillin and streptomycin, except for prior to high-performance liquid chromatography (HPLC) analysis when DMEM with 1 mM glucose was substituted for RPMI-1640. A-498 (VHL-), ACHN (VHL+), Caki-1 (VHL+) and 786-O (VHL-) cells were purchased from, and validated by, ATCC. These cells were cultured in DMEM with 1 mM glucose, 10% FBS, penicillin and streptomycin. Renal primary proximal tubule epithelial cells (RPTEC) were purchased from and validated by Lonza, and were cultured in renal epithelial cell growth medium (Lonza).

### Kynurenine metabolites cell growth assays

Kynurenine and 3-HK (100 mM) were prepared in 1M HCl, 3-HAA (50 mM) was prepared in 1M HCl, and kynurenic acid and quinolinic acid (100 mM) were prepared in 1M NaOH and stored at -20°C. Equal volumes of 1M HCl and 1M NaOH, with or without each metabolite were added to media. The media was changed the day following plating to medium supplemented with metabolites for a further three days, with medium changed daily.

### Methylthiazolyldiphenyl-tetrazolium bromide (MTT) assay

Cell viability was measured using MTT as previously described [34]. All values were expressed relative to control wells set at 100%.

### IDO inhibitor cell growth assays

1-L-MT and 1-D-MT (20 mM) were prepared in 0.1N HCl, 50% DMSO in sterile PBS. MTH-trp (100 mM) was prepared in DMSO. Human RCC and RENCA cells were treated with inhibitors or DMSO for three days. Cell number was determined using a methylene blue assay as described [35].

### Immunoblotting

Immunoblotting with primary antibodies (rabbit polyclonal anti-human IDO1, 1:1000, Cell Signaling Technology, Danvers, MA; monoclonal rat anti-mouse IDO1, 1:500, BioLegend, San Diego, CA; monoclonal rabbit anti-β-actin, 1:2000, Cell Signaling Technology; monoclonal mouse anti-β-actin, 1:4000, Sigma-Aldrich; rabbit monoclonal anti-vinculin, 1:1000, Cell Signaling Technology) was detected by chemiluminescence as previously described [36]. Alternatively, fluorescence was quantified on the Odyssey Imaging System (Li-Cor BioSciences, Lincoln, Nebraska).

### Tryptophan and kynurenine quantification

Human RCC and RENCA cells were plated at sub-confluence in 6-well plates and treated with murine IFNγ or human IFNγ and/or MTH-trp (100 μM) for 72 h prior to the collection of medium, its centrifugation (1000 g, 5 min), and storage of supernatant at -80°C. Extraction and quantitation of these metabolites was as described [37].

### Immunohistochemistry

Tissue from ccRCC tumors and adjacent normal kidneys were archived following IRB approval at UC Davis Department of Pathology. Paraffin sections (4 μm) of formalin-fixed tissue were stained for IDO1 using heat-induced antigen retrieval in citrate buffer (pH 6.0) and anti-human IDO1 (Cell Signaling Technology), followed by Mach 2 Rabbit HRP-Polymer (BioCare Medical, Concord, CA) and ImmPACT diaminobenzidine peroxidase substrate (Vector Laboratories, Burlingame, CA). Immunohistochemistry for CD68 was done using heat-induced antigen retrieval at pH 9 (Dako, Carpinteria, CA), mouse monoclonal anti-CD68 (Leica Biosystems, Newcastle Upon Tyne, UK) and Mouse-on-canine HRP-polymer (BioCare Medical) prior to detection with NovaRed (Vector Laboratories). A pathologist (F.U.) scored IDO staining using a subjective scale of 0 (no IDO staining) to 3 (20-40% of cells were stained) without assigning a grade to the tumors in order to minimize possible bias of the IHC analysis. No more than 40% of all cells in any slide stained positive. Staining of CD68 was scored as a percentage of interstitial cells that were positive with a score 0 (no staining) to 5 (40-50% positive stained interstitial cells).

### Quantitative PCR (qPCR)

Total RNA was extracted from tissue or cells using Trizol (Invitrogen) and its integrity confirmed by gel electrophoresis. Total RNA (0.5 μg) was reverse transcribed in 20 μl using MultiScribe reverse transcriptase (Thermo Fisher Scientific; 50U). Primers for qPCR (Table 3) were designed using Primer Blast (http://www.ncbi.nlm.nih.gov/tools/primer-blast/) to be in different exons and not amplify non-specific cDNA or gDNA. This was confirmed by performing PCR on reverse transcription negative control reactions performed in the absence of reverse transcriptase. The tryptophan-2,3-dioxygenase (*TDO2*) and mouse glyceraldehyde-3-phosphate dehydrogenase (*Gapdh)* PCR products were confirmed by sequencing. Reference genes for mouse samples were 18S ribosomal RNA (*Rn18S*) and *Gapdh* (Table 3). Reference genes for human samples were cyclophilin A (*PPIA*) and ribosomal protein S13 (*RPS13*) as described by Dupasquier *et al*., [16; Table 3]. The cDNA was diluted (1:4 or 1:50) before 1 μl was analyzed using SYBR green PCR master mix (Applied Biosystems, Foster City, CA) with 0.25 μM primers on a ViiA™ 7 Real-Time PCR System (Applied Biosystems). Cycling conditions were 50°C for 2 min, 95°C for 10 min then 40 cycles of 95°C for 15 s and 1 min at 60, 62, 63 or 64°C (Table 3). Each qPCR run included a no-template control containing all reagents except cDNA. Standard curves were prepared using 6 to 7 five-fold serial dilutions of either mouse or human liver cDNA. One standard curve was used for all qPCR plates within an experiment. All standard curves had a linear regression coefficient of determination of at least 99.4%. The mRNA or rRNA levels in each mouse sample were calculated from Ct values using a standard curve. The relative mRNA levels in each human sample were calculated from Ct values using the delta-delta Ct method relative to an average of the two housekeeping Ct values.

**Table 3:** PCR primers used for quantitative PCR

Accession Number	Gene	Forward and Reverse Primers (5′-3′)	Tm^1^ (°C)	E^2^ (%)
NM_005651	h*TDO2*	GCACTTCAGGGAGCATTGAT TCACTCACAGTTGATCGCAG	64	103
	h*IDO1* [13]	GGTCATGGAGATGTCCGTAA ACCAATAGAGAGACCAGGAAGAA	62	97
NM_019911	m*Tdo2*	ATGGCTGGAAAGAACACCTG CATCAAACAAGCAGAGCAGC	63	79
	m*Ido1* [14]	GTACATCACCATGGCGTATG CGAGGAAGAAGCCCTTGTC	60	88
NM_001289726	m*Gapdh*	TGATGGGTGTGAACCACGAG AAGTCGCAGGAGACAACCTG	63	53
	*Rn18S* [15]	ACGGCTACCACATCCAAGGA CCAATTACAGGGCCTCGAAA	60	90
	*PPIA* [16]	ACCGCCGAGGAAAACCGTGTA TGCTGTCTTTGGGACCTTGTCTGC	64	95
	*RPS13* [16]	TCGGCTTTACCCTATCGACGCAG ACGTACTTGTGCAACACCATGTGA	64	101

1 Annealing and extension temperature.

2 Primer pair efficiency (E).

### qPCR data normalization

Reference gene expression was normalized within an experiment so the average expression across all samples for each reference gene was the same. Normalization of gene expression relative to two reference genes was completed according to the formula [38]:

$$Y_{i}=\frac{Q_{\text{Ti}}}{\sqrt[{2}]{Q_{R1i}\times Q_{R2i}}}$$

where Y is the normalized tryptophan-2,3-dioxygenase (*TDO2)* or *IDO1* gene expression for tissue/cell line i, Q_Ti_ is *TDO2* or *IDO1* mRNA quantity and Q_R1i_ and Q_R2i_ are reference gene quantities.

### Statistical analysis

All data are expressed as mean ± standard error of the mean. Data for relative tumor growth and tumor weights were normalized using an optimal Box-Cox power transformation where necessary to satisfy Levene's test for homogeneity of variance and analyzed in SAS 9.4 using the mixed procedure to fit a mixed linear model (SAS Institute, Inc., Cary, NC). Mouse was the experimental unit with treatment and RENCA cell passage number being fixed effects, while cage was considered a random effect. For all other experiments, two-tailed Students *t*-tests were used to compare means. Means were considered different at *P* < 0.05.

## SUPPLEMENTARY MATERIAL TABLES


