# Supplementary material for: Inhibiting tryptophan metabolism enhances interferon therapy in kidney cancer
Source: Oncotarget. 2016 Aug 27;7(41):66540–57. doi: 10.18632/oncotarget.11658 (PMC5341819; doi:10.18632/oncotarget.11658)
Supplement: Supplementary file 1 [file oncotarget-07-66540-s001.pdf]

Inhibiting tryptophan metabolism enhances interferon therapy in kidney cancer

Supplementary Material

Table S1. PCR primers used for quantitative PCR.

Accession Number	Gene	Forward and Reverse Primers (5'-3')	Tm[1] (°C)	E[2] (%)
NM_005651	h*TDO2*	GCACTTCAGGGAGCATTGAT<br>TCACTCACAGTTGATCGCAG	64	103
	h*IDO1*[3]	GGTCATGGAGATGTCCGTAA<br>ACCAATAGAGAGACCAGGAAGAA	62	97
NM_019911	m*Tdo2*	ATGGCTGGAAAGAACACCTG<br>CATCAAACAAGCAGAGCAGC	63	79
	m*Ido1*[4]	GTACATCACCATGGCGTATG<br>CGAGGAAGAAGCCCTTGTC	60	88
NM_001289726	m*Gapdh*	TGATGGGTGTGAACCACGAG<br>AAGTCGCAGGAGACAACCTG	63	53
	Rn18S[5]	ACGGCTACCACATCCAAGGA<br>CCAATTACAGGGCCTCGAAA	60	90
	PPIA[6]	ACCGCCGAGGAAAACCGTGTA<br>TGCTGTCTTTGGGACCTTGTCTGC	64	95
	RPS13[6]	TCGGCTTTACCCTATCGACGCAG<br>ACGTACTTGTGCAACACCATGTGA	64	101

[1]Annealing and extension temperature.

[2]Primer pair efficiency (E).

[3]Reisenberg *et al*. [1]

[4]Uyttenhove *et al*. [2]

[5]Rowson-Hodel *et al*. [3]

[6]Dupasquier *et al.* [4]

<u>qPCR Methods</u>

Total RNA was extracted from tissue or cells using Trizol (Invitrogen) and its integrity confirmed by gel electrophoresis. Primers for qPCR (Table S1) were designed using Primer Blast (http://www.ncbi.nlm.nih.gov/tools/primer-blast/) to be in different exons and not amplify non-specific cDNA or gDNA. This was confirmed by performing

PCR on reverse transcription negative control reactions performed in the absence of reverse transcriptase. The tryptophan-2,3-dioxygenase (*TDO2*) and mouse glyceraldehyde-3-phosphate dehydrogenase (*Gapdh)* PCR products were confirmed by sequencing. Reference genes for mouse samples were 18S ribosomal RNA (*Rn18S*) and *Gapdh* (Table S1). Reference genes for human samples were cyclophilin A (*PPIA*) and ribosomal protein S13 (*RPS13*) as described by Dupasquier *et al*., [4; Table S1]. Total RNA (0.5 μg) was reverse transcribed in 20 μl using MultiScribe reverse transcriptase (Thermo Fisher Scientific; 50U). The resulting cDNA was diluted (1:4 or 1:50) before 1 μl was analyzed using SYBR green PCR master mix (Applied Biosystems, Foster City, CA) with 0.25 μM primers on a ViiA™ 7 Real-Time PCR System (Applied Biosystems). Cycling conditions were 50°C for 2 min, 95°C for 10 min then 40 cycles of 95°C for 15 s and 1 min at 60, 62, 63 or 64°C (Table S1). Each qPCR run included a no-template control containing all reagents except cDNA. Standard curves were prepared using 6 to 7 five-fold serial dilutions of either mouse or human liver cDNA. One standard curve was used for all qPCR plates within an experiment. All standard curves had a linear regression coefficient of determination of at least 99.4%. The mRNA or rRNA levels in each mouse sample were calculated from Ct values using a standard curve. The relative mRNA levels in each human sample were calculated from Ct values using the delta-delta Ct method relative to an average of the two housekeeping Ct values.

Table S2. Immunohistochemistry staining score of RCC and adjacent normal kidney tissue.

Antibody	IDO1[3]	IDO1	IDO1	CD68[4]
Tissue	Endothelial cells	Neoplastic cells	Interstitial cells	Interstitial cells
Kidney Neg[1]	0	0	0	n/a
Kidney-1	0	0	0	0.5
Kidney-2	0	0	0	0.5
Kidney-3	0	0	0	1
Grade 2[2] Neg	0	0	0	0
Grade 2-1	1	0	1	2.5
Grade 2-2	3	1	1	3
Grade 2-3	2	1	1	2
Grade 3 Neg	0	0	0	0
Grade 3-1	2	0	1	4
Grade 3-2	2	0	1	5
Grade 3-3	3	0	1	2.5

[1]Neg=secondary antibody only

[2]Grade of renal cell carcinoma (RCC)

[3]Score for IDO1: 0=no staining, 1=1-10%, 2=10-20%, 3=20-40% staining, respectively

[4]Score for CD68: 0=no staining, 1=1-10%, 2=10-20%, 3=20-30%, 4=30-40%, 5=40-50% positive interstitial cells

REFERENCES

1. Riesenberg R, Weiler C, Spring O, Eder M, Buchner A, Popp T et al. Expression of indoleamine 2,3-dioxygenase in tumor endothelial cells correlates with long-term survival of patients with renal cell carcinoma. Clin Cancer Res. 2007; 13(23):6993-7002.
2. Uyttenhove C, Pilotte L, Theate I, Stroobant V, Colau D, Parmentier N et al. Evidence for a tumoral immune resistance mechanism based on tryptophan degradation by indoleamine 2,3-dioxygenase. Nat Med. 2003; 9(10):1269-74.
3. Rowson-Hodel AR, Manjarin R, Trott JF, Cardiff RD, Borowsky AD, Hovey RC. Neoplastic transformation of porcine mammary epithelial cells in vitro and tumor formation in vivo. BMC Cancer. 2015; 15:562.
4. Dupasquier S, Delmarcelle AS, Marbaix E, Cosyns JP, Courtoy PJ, Pierreux CE. Validation of housekeeping gene and impact on normalized gene expression in clear cell renal cell carcinoma: critical reassessment of YBX3/ZONAB/CSDA expression. BMC Mol Biol. 2014; 15:9.
